# MiRNAs in the Peri-Implantation Period: Contribution to Embryo–Maternal Communication in Pigs

**DOI:** 10.3390/ijms21062229

**Published:** 2020-03-23

**Authors:** Monika M. Kaczmarek, Joanna Najmula, Maria M. Guzewska, Emilia Przygrodzka

**Affiliations:** 1 Institute of Animal Reproduction and Food Research, Polish Academy of Sciences, 10-748 Olsztyn, Poland; j.najmula@pan.olsztyn.pl (J.N.); m.guzewska@pan.olsztyn.pl (M.M.G.); 2 University of Nebraska Medical Center, Omaha, NE 68198, USA; emilia.przygrodzka@unmc.edu

**Keywords:** miRNAs, extracellular vesicles, corpus luteum, conceptus, endometrium, pregnancy, embryo–maternal communication, maternal recognition of pregnancy, implantation

## Abstract

MicroRNAs (miRNAs) constitute a large family of noncoding RNAs, approximately 22 nucleotides long, which function as guide molecules in RNA silencing. Targeting most protein-coding transcripts, miRNAs are involved in nearly all developmental and pathophysiological processes in animals. To date, the regulatory roles of miRNAs in reproduction, such as fertilization, embryo development, implantation, and placenta formation, among others, have been demonstrated in numerous mammalian species, including domestic livestock such as pigs. Over the past years, it appeared that understanding the functions of miRNAs in mammalian reproduction can substantially improve our understanding of the biological challenges of successful reproductive performance. This review describes the current knowledge on miRNAs, specifically in relation to the peri-implantation period when the majority of embryonic mortality occurs in pigs. To present a broader picture of crucial peri-implantation events, we focus on the role of miRNA-processing machinery and miRNA–mRNA infarctions during the maternal recognition of pregnancy, leading to maintenance of the corpus luteum function and further embryo implantation. Furthermore, we summarize the current knowledge on cell-to-cell communication involving extracellular vesicles at the embryo–maternal interface in pigs. Finally, we discuss the potential of circulating miRNAs to serve as indicators of ongoing embryo–maternal crosstalk.

## 1. Introduction

Early pregnancy in domestic animals, such as pigs, begins with the period of development of the embryo(s) in the oviduct to reach the blastocyst stage in the uterus. After hatching from the *zona pellucida*, the blastocyst elongates to increase its physical contact area with the endometrium and starts an intensive dialogue with the uterus, establishing the foundation for a successful pregnancy. As in other mammals, conceptus development requires the uterus and is concomitant with a maternal recognition of pregnancy (days 10–13 post-estrus) via embryonic signaling to maintain the corpus luteum (CL) function (i.e., progesterone (P4) production) as well as the onset of implantation (days 14–19) and further placenta formation.

In pigs, two main embryonic signals—estradiol-17beta (E2) and prostaglandin E2 (PGE2)—prevent regression of the CL, which occurs on days 13–14 of the estrous cycle. CL rescue is achieved by a paracrine action of the conceptus on the endometrium and the luteal tissue to stop the beginning of luteolysis [1]. Reciprocal communication between the preimplantation conceptus not only results in maintaining P4 production by the ovaries but also sets up the environment for successful embryonic development and implantation.

Uterine receptivity for conceptus elongation and implantation develops during each cycle in response to P4 and is modified in response to products secreted by the conceptus and endometrium sensed by the competent embryo. Preparation of the uterus synchronized with the development of the embryo is necessary to establish proper communication between the embryo and the mother during early pregnancy. Establishing firm connections between the endometrium and the embryo requires a specific mutual dialogue achieved through the secretion of steroid hormones as well as a number of different agents, such as growth factors, prostanoids, and cytokines [2,3]. Importantly, the growing conceptus has to communicate with the endometrium not only to appose, attach, and implant, but also to sustain favorable conditions for its own growth. Recently, microRNAs (miRNAs) have emerged as new players in the fine-tuning of the endometrial milieu, embryo development, and implantation in mammals via posttranscriptional gene regulation mechanisms.

In this review, we summarize recent progress in miRNA-mediated regulations of the peri-implantation period in pigs and highlight the significance of miRNAs in different aspects of successful pregnancy outcomes. First, we discuss the importance of miRNA-processing machinery during early pregnancy. Second, we focus on the dynamics of peri-implantation events, looking at miRNAs at the embryo–maternal interface and in the CL during pregnancy. Furthermore, we summarize the role of miRNA transport at the embryo–maternal interface involving extracellular vesicles (EVs). Finally, we discuss an intriguing characteristic of miRNAs—their presence in circulation—and provide a set of conclusive remarks to guide future studies.

## 2. Overview of MiRNA Biogenesis and Function

MiRNAs are a class of endogenous, small non-coding RNAs, with an average length of 22 nucleotides. Slightly over half of all currently identified miRNAs are intergenic, located in noncoding transcription units, while the remaining miRNAs are intragenic, processed mostly from introns and relatively few exons of protein-coding genes [4,5]. In addition, miRNAs can occur in clusters, which means that two or more miRNAs are simultaneously transcribed from adjacent miRNA genes [6]. This intriguing family of regulatory molecules is mostly transcribed by RNA polymerase II from DNA sequences into primary miRNAs (pri-miRNAs), containing a local stem–loop structure that encodes miRNA sequences in the arm of the stem. Pri-miRNAs are processed by a DROSHA/DGCR8 microprocessor complex into 70–100 nt hairpin/stem–loop precursors (pre-miRNAs). Once exported from the nucleus by exportin-5 (XPO5), pre-miRNAs are cleaved by DICER1 (RNase III endonuclease) to produce RNA duplexes approximately 22 nt long, one strand of which is selected and incorporated into the effector complex, RNA-induced silencing complex (RISC) [7,8]. DICER1 and its protein partners, HIV-1 transactivation response (TAR), RNA-binding protein 2 (TARBP2), a trinucleotide repeat containing 6A (TNRC6A), and members of the Argonaute (AGO1–4) family, are the main components of this complex. RISC binds to the target mRNA in a sequence-specific manner and induces mRNA cleavage, translational inhibition, or cleavage-independent mRNA degradation [9,10]. Although the aforementioned canonical pathway accounts for the production of most miRNAs, it has also been shown that alternative (non-canonical) pathways for miRNA biogenesis exist, which bypass part of the abovementioned biogenesis steps [11]. In most cases, miRNAs interact with the 3′ UTR of target mRNAs to suppress expression [12]. However, miRNA interactions with other regions, such as the 5′ UTR or coding sequence, have also been reported [13]. While a single miRNA may target many genes, the expression of a particular gene can be modulated by multiple miRNAs [14]. Interestingly, miRNAs belonging to the same genomic cluster can share targets between specific signaling pathways [6], which consequently can enhance miRNAs’ mediated effects on biological functions.

## 3. MiRNA Biogenesis at the Embryo-Maternal Interface

Several reports have suggested that the disrupted expression of genes involved in miRNA synthesis or transport, mainly DICER1 and AGO2, may have a serious effect on reproductive functions or embryo development, most likely due to altered miRNA levels [15,16]. The importance of DICER1 expression in early embryos has already been reported in animals, including domestic livestock such as pigs [17] and cattle [18]. Notably, knockout of Dicer1 in mouse causes morphologic abnormalities and stunted growth in embryonic day (E) 7.5 embryos and lethality by E11.5 [19], clearly showing its crucial role in development. Among others, the loss of Dicer in the mouse reproductive tract (Dicer Amhr2-cKO) compromised uterine development, leading to pregnancy loss following the wild-type embryo transfer [20]. Interestingly, simultaneous estrogen and P4 treatment in mice increased the uterine expression of *DROSHA, DGCR8, XPO5*, and *DICER1* [21], suggesting involvement of steroid hormones in miRNA biogenesis.

Recently, pregnancy stage-dependent expression profiles of genes involved in miRNA synthesis and transport were demonstrated in porcine conceptuses, trophoblasts, and endometria [22,23]. Uterine glands and luminal epithelia were the only sites of DICER1 and AGO2 immunoreactivity, where miRNA biogenesis seems likely to occur during early pregnancy in pigs. Remarkably, *AGO4* and *DGCR8* endometrial expression significantly increased on day 16 of pregnancy, concomitantly with a slight increase of *DICER1* and *AGO3*. Conceptuses, however, showed increased expression of *XPO5*, *DICER1*, *TNRC6A,* and AGO2 on days 11–12, when porcine blastocysts undergo rapid and radical morphological changes from spherical, through tubular, to filamentous forms, accompanied by increasing transcriptomic activity and E2 secretion [24,25]. Characteristic, dynamic gene expression patterns involved in miRNA biogenesis and transport observed in endometria and developing conceptuses on days 10–20 of pregnancy point at the crucial involvement of miRNAs in the regulation of peri-implantation events in pigs.

## 4. MiRNAs at the Embryo—Maternal Interface

The key phenomena of porcine early pregnancy are the prolonged embryo migration throughout the uterus and the following epitheliochorial placentation when maternal and fetal layers lay in close contact without invading each other [26]. At this stage, a great variety of molecules is exchanged between both parties [3]. Consequently, dramatic transcriptome changes can be observed, and thousands of genes are differentially expressed at this time in both contacting tissues [27,28,29,30]. Those very time-specific transcriptomic changes demand precise regulations that, among others, are driven by miRNAs.

To date, numerous miRNAs have been detected at different stages of porcine embryo development, ranging from zygotes and early embryos [17,31], through peri-implantation embryos and its trophoblasts [22,32,33,34], to placentas on day 90 of gestation ([35,36]; see examples in Table 1).

Comparisons of 8-cell embryos and day 7 blastocysts revealed 13 miRNAs, differentiating both developmental stages. Their potential targets were overrepresented in Gene Ontology categories, such as cell motion and migration, helicase activity, chromatin organization, transcriptional activity, and cellular component enrichment of Golgi cis cisternae—processes that are crucial during the formation of blastocysts, when cells rapidly proliferate, differentiate, and migrate to create the inner cell mass and trophectoderm [17]. Later stages of porcine embryo development were studied using high-throughput sequencing or microarrays and revealed hundreds (368 and 220, respectively) of miRNAs expressed during the peri-implantation period of pregnancy [22,34]. Both studies found that the identified miRNAs are potential regulators of important processes for embryo development and maternal-embryo interaction (e.g., TGF-beta and p53 signaling, cellular motility, and gonadotropin-releasing hormone signaling).

The miRNAome of the endometrium during pregnancy was widely characterized by microarrays [33,37] and sequencing [23]. MiRNAs differentially expressed between days 15 and 50 of gestation were mostly involved in endometrial remodeling (e.g., cell proliferation, migration, apoptosis, cytoskeleton organization, and blood vessel development) and cell communication (e.g., response to hormone stimulus, and cell-to-cell and cell-to-matrix adhesion) [37]. Using different methods, the set of miRNAs worth noticing is consistently shown by two groups, as differentially expressed between day 20 pregnant vs. cyclic endometrium: miR-27a, miR-30d, miR-205, and miR-574 [23,33].

Intriguingly, porcine endometria [23] as well as blastocysts [38] are abundant not only in canonical miRNAs but also isomiRs—widely reported variants of miRNAs—which have been proven to work cooperatively [43]. Many miRNAs can be co-expressed with their isomiRs and/or other members of the family (e.g., 23a/b, 148a/b in the porcine endometrium), resulting in wider or stronger biological effects. Although the role of a vast reservoir of isomiRs in reproductive tissues remains unclear, it was proven that transfection with either miR-140-3p, its isomiR, or both simultaneously added significantly downregulated putative target gene expression (i.e., *ACVR2B, KCNMA1, SIRT1, LIF*) in porcine stromal cells [23].

The key molecules of maternal recognition of pregnancy in pigs are estrogens (mainly E2) secreted by conceptuses during rapid elongation [44]. For years, it has been known that disrupted estrogen exposure can lead to pregnancy abnormalities [45,46]. Interestingly, uterine miRNA biogenesis components were shown to be under steroidal regulation in rodents [21]. Our studies suggested a similar regulatory relationship in the porcine endometrium [23]. However, neither endometrium [47] nor blastocyst [38] miRNAome was shown to be affected on day 10 of pregnancy by E2 administered daily to sows in different doses, even though its concentrations significantly increased in the endometrium.

Differences in endometrial and/or conceptus miRNA expression can be strain-specific and related to the arrest of embryo development and litter size. The peri-implantation endometria of Yorkshire and Chinese Meishan breeds that vary in litter size were reported to differ in miRNA expression. MiRNAs differentially expressed between both stains around day 12 of pregnancy were involved in the regulation of p53 and Wnt signaling pathways, which are both crucial for proper implantation in pigs [48]. When miRNA expressions of trophoblasts of healthy and arrested embryos were compared, only 3 (miR-15b-5p, miR-18a, and miR-222) out of 11 detected miRNAs were differentially expressed, similar to the matching endometrial biopsies (miR-20b, miR-17-5p, and miR-18a) [32]. Indeed, miR-18a has already been associated with human fetal macrosomia [49] and preeclampsia [50]. Recently, it was shown that a single-nucleotide polymorphism (SNP) within a miR-18a binding site within CDC42 3′UTR lowers the ability of this miRNA to regulate and influence litter size by affecting oocyte maturation and placental development in pigs [51]. In addition, an SNP within a miR-27a gene was also found to be associated with pregnancy outcome. Specifically, a T/C mutation in a coding sequence of miR-27a was associated with litter size, and, as suggested by the authors, could serve as a molecular marker for litter size in pig breeding programs [52].

Early pregnancy events are accompanied by intensive changes in the mother’s immune system. Top-ranked processes affected by differentially expressed miRNAs in trophoblasts between days 12 to 20 of pregnancy indicate their role in inflammatory processes and cell-mediated immune response. The number of inflammatory mediators can be potentially targeted by miRNAs detected in endometria [23] and trophoblasts [22]. Eventually, downregulation of LIF and its receptor in endometrial luminal epithelial cells by miR-125b was validated *in vitro* [22]. In the endometrium following insemination and during different stages of early pregnancy, there are noticeable variations in the distribution of immune cells [53]. Analysis of miRNAs in the lymphocytes of endometria in days 20 and 50 of gestation revealed a set of miRNAs involved in immune cell development at the fetal–maternal interface. Interestingly, miR-150, miR-296-5p, and miR-19a were differentially expressed in endometria with arrested and healthy embryos on day 20. Since most of these miRNAs are involved in angiogenesis and immune cell development, the authors suggested that arrested conceptuses try to compensate for their potential lack of angiogenesis and/or other processes essential for survival [32].

## 5. MiRNAs Carried by EVs at the Embryo–Maternal Interface

For the first time, small vesicles along the interface between the porcine endometrium and developing conceptuses were detected on day 16 of pregnancy by electron microscopy in the early 1990s of the last century [54]. This observation was the first hint that EVs can be active mediators of embryo–maternal communication during early pregnancy in pigs. To date, EVs have been isolated from different body fluids in different species, including porcine seminal plasma [55] and uterine lumen flushings [22]. Recently, intensive secretion of EVs by endometrial and trophoblast cells was detected during the peri-implantation period in pigs. A large population of EVs of different sizes was observed in the lumen of uterine glands, along with a number of multivesicular bodies located in the cytoplasm of luminal and glandular epithelia as well as trophoblast cells [56]. This is consistent with the current knowledge that EVs can be secreted by most cells in physiological and pathological conditions, in response to different stimuli and changes in the extracellular environment [57].

EVs are a heterogeneous population of biological particles that are surrounded by a double phospholipid membrane. EVs have been divided into two groups, according to their size: microvesicles/ectosomes (100–1000 nm), budding directly out from the plasma membrane; and exosomes (30–100 nm), intraluminal vesicles created during maturation of multivesicular bodies released from the originating cell via exocytosis [58]. The endosomal sorting complex required for transport (ESCRT) catalyzes the formation of multivesicular bodies [59]. The ESCRT complex is composed of approximately 30 proteins that assemble into four complexes, ESCRT-0, -I, -II, and -III. Subunits of ESCRT recognize ubiquitylated cargo, deform endosomal membranes, and catalyze intraluminal vesicle formation with sorted cargo [60]. EVs have the ability to carry different molecules such as proteins, DNA, various RNA forms, including miRNAs, and other cellular and membrane components (e.g., tetraspanins CD63, CD9), contributing to intercellular communication [61]. Trafficking routes in the generation of EVs also involve RAS-related protein (RAB) GTPases, master regulators of intracellular membrane traffic [58]. To date, the organization and activity of the ESCRT complex and vesicle trafficking have not yet been studied during early pregnancy in pigs. Our preliminary results suggest that reproductive status (pregnant, non-pregnant) affects the expression of ESCRT and RAB family members in the endometrium and trophoblast during peri-implantation in pigs [62].

**Figure 1:**
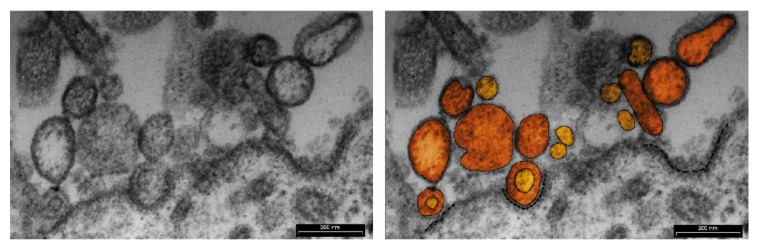
Uptake of EVs via clathrin-maintained endocytosis by trophoblast cell on day 16 of pregnancy. Two types of EVs internalized by endocytosis are located in the extracellular space. Orange–large vesicles ectosomes, and yellow–small exosomes. Clathrin-coated pits are marked by black dashed line.

The main functions of EVs depend on their ability to interact with recipient cells and to deliver their content. Each cell type may use different pathways of EV uptake, but generally, uptake seems to be a complex and cooperative process involving a combination of different endocytosis pathways. EVs can use receptors to enter target cells and stimulate them via a ligand–receptor interaction, guided by adhesion molecules such as integrins or tetraspanins [58]. Clathrin-dependent endocytosis controls the internalization of many receptors and signal transduction proteins implicated in many key cellular functions such as growth, differentiation, migration, or proliferation [63]. Morphologically, the first stage of clathrin-dependent endocytosis starts through the membrane remodeling process. The membrane deforms into clathrin-coated pits, and invaginated vesicles detach after cargo selection and clathrin uncoating to move along microtubules in the cytoplasm. We observed this mechanism using transmission electron microscopy during the peri-implantation period in pigs. On day 16 of pregnancy, at the apical site of trophoblast cells, we confirmed the uptake of EVs via clathrin-maintained endocytosis (Figure 1). Notably, this pathway of EVs uptake has not yet been analyzed in relation to the cell-to-cell commutation at the embryo–maternal interface during early pregnancy in pigs and other species.

As described before, EVs can horizontally transfer mRNA and miRNA to target cells, which then trigger the translation of functional proteins or induce gene silencing [64]. The involvement of endometrial EVs carrying unique miRNAs in embryo-maternal crosstalk at implantation has already been suggested for several species, including pigs [22,39], sheep [65], and humans [66]. The first discovery of EVs that carry miRNAs in the uterine environment of pigs on days 14 and 16 of pregnancy reinforced the idea of the horizontal transfer of miRNAs between the endometrium and conceptuses in order to support growth and development, implantation, and placenta formation. Indeed, miR-125b identified in uterine lumen-derived EVs delivered to day 12 endometrial luminal epithelial cells, the first site of signal exchange between the mother and conceptus, affected the expression of genes important during pregnancy, such as *LIF* and *LIFR* [22]. In addition, EVs secreted *in vitro* by porcine trophectoderm cells were shown to carry miRNAs (e.g., miR-16, miR-17-5p, miR-20a) and stimulate proliferation of porcine aortic endothelial cells, indicating the possibility of EVs–miRNA mediated effects on the regulation of placental angiogenesis [39]. All of the abovementioned data clearly showed that EVs are active mediators of two-way communication between the embryo and the mother during the peri-implantation period.

## 6. MiRNAs in Pregnant Corpus Luteum

The fundamental role of miRNAs in ovarian function was proved by knockout of DICER, which inhibited follicle growth, and reduced the ovulation rate and faulty oocyte development [16,67,68]. Further studies showed a number of differentially expressed miRNAs in the bovine and ovine CL during development, regression, or its rescue during pregnancy [69,70,71,72,73,74]. Interestingly, a greater amount of miRNAs was found in the mature than in the developing CL, indicating their potential role in the maintenance of luteal function. Nevertheless, not many studies have provided information on the specific roles of selected miRNA–mRNA interactions on the function of luteal cells, while only a few have shown changes in the expression of miRNAs occurring in the CL during early pregnancy [40,72].

Recently, 14 differentially expressed miRNAs were identified in porcine CL collected right after maternal recognition of pregnancy (day 14 of pregnancy) and luteolysis (day 14 of the estrous cycle) [40]. Overall, seven miRNAs were upregulated (e.g., miR-21a-3p, miR-345-3p, miR-371-5p), and seven miRNAs were downregulated (e.g., miR-181a, miR-532-3p, miR-99b) in the porcine CL on day 14 of the estrous cycle compared to the corresponding day of pregnancy. Interestingly, among miRNA targets upregulated during pregnancy, genes encoding known regulators of luteolysis were found (e.g., *EDN1*, *FOS*, *JUN*, *PTGS2*, *ESR2*) [75,76,77,78], while miRNAs elevated in the CL during luteolysis could target genes associated with luteal function maintenance (e.g., *PGR*, *VEGFR1*, *CREB*, *PTGER2*). Further *in silico* analysis suggested that miRNAs highly expressed in the CL during early pregnancy can be involved in the cell cycle, cell death and survival, cellular development, growth, and proliferation—events characterizing the time when a decision is made either to regress or maintain the luteal function. Notably, the expression of miRNAs was examined in the whole luteal tissue. Therefore, the aforementioned biological processes can be potentially regulated by differentially expressed miRNAs in various cells forming the porcine CL, such as the endothelial cells, immune cells, or fibroblasts, and not only the luteal cells. Other studies performed on the bovine CL identified 15 miRNAs differentially expressed on day 18 of pregnancy vs. the corresponding day of the estrous cycle, while among predicted targets of these miRNAs, genes involved in immune-related events and apoptosis were found [72].

Previously, the high expression of two clusters, miR-183-96-182 and miR-212-132, was noticed in the luteal vs. follicular tissue of cows, while miR-96 was found to be an important regulator of steroidogenesis and cell survival [74]. In pigs, miRNAs upregulated in the CL of pregnant animals belong to three independent clusters: miR-99b, miR-532, and miR-181a [40]. Again, among targets of miRNAs occurring in the same cluster (i.e., miR-532 and miR-99b), crucial regulators of luteolysis were found, including, for example, *EDN1*, *FOS*, *JUN*, *NR4A1*, *OXTR*, and *PTGS2* [75,76,78]. Interestingly, identified clusters are conserved or broadly conserved among different animal species and humans; thus, it is possible that some of them can support luteal function in other species as well, especially since miRNAs belonging to cluster miR-99b were previously detected among mostly abundant miRNAs in the mature bovine CL [73].

**Figure 2:**
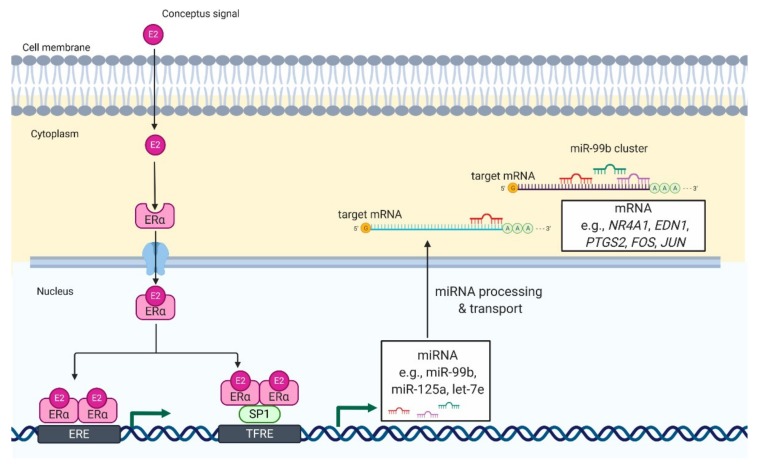
Proposed model of E2-mediated regulation of miRNA expression in luteal tissue during early pregnancy in pigs. E2 enters the luteal cell and binds with its genomic receptor, ER*α*, located in the cytoplasm. It leads to formation of ER*α* homodimers and their translocation to the nucleus. In the nucleus, ERα homodimers bind to (1) an estrogen-responsive element (ERE) or (2) transcription factor SP1, which binds to transcriptional factor responsive elements (TFRE) in the promoter region of miR-99b cluster. As a result, there is an increase in the expression of miRNAs belonging to miR-99b cluster (miR-99b, miR-125a, and let-7e), which can target mRNA involved in luteolysis including NR4A1, nuclear receptor subfamily 4 group A member 1; EDN1, endothelin 1; FOS, fos proto-oncogene; JUN, jun proto-oncogene. That mechanisms support the function of luteal cells and allow continuation of progesterone production required for pregnancy establishment and maintenance.

Luteotrophic and/or luteolytic factors were suggested to be effective modulators of miRNA expression [74,79]. In pigs, incubation of luteal tissue slices with E2 stimulated the expression of miRNAs belonging to the miR-99b cluster [40]. Further, bioinformatics analysis showed multiple SP1 and ESR1 binding sites in the promoter of the miR-99b cluster. SP1 is a zinc finger transcription factor that acts synergistically with ESR1, one of the genomic receptors of estradiol, in the regulation of gene transcription. Therefore, we suggest that E2 increases the expression of miR-99b by the ESR1-SP1 pathway in the CL during early pregnancy. Remarkably, the aforementioned effects of E2 on the expression of the miRNA-99b cluster were concomitant with the inhibitory effect of E2 on the expression of *NR4A1* and *AKR1C1* in luteal tissue slices [40]. NR4A1 is encoded by immediate-early response genes, which can induce apoptosis or elevate transcription of AKR1C1, an enzyme responsible for the metabolism of progesterone [80,81]. NR4A1 was identified as a marker of luteolysis in the CL of cows and rats [80,81,82]. Our further studies showed that the transfection of luteal tissue slices with mimics of miRNAs belonging to the miR-99b cluster decreases the expression of NR4A1 and AKR1C1, and increases the production of progesterone by luteal tissue slices of pigs [40]. Therefore, it seems likely that E2 can induce the expression of the miR-99b cluster in porcine CL during the peri-implantation period, leading to decreased expression of genes involved in luteolysis (Figure 2).

## 7. Circulating MiRNAs during Early Pregnancy

MiRNAs have been detected in several extracellular fluids, including plasma and serum [83,84], showing great stability due to their resistance to endogenous ribonucleases [85]. Their stability in blood might also be related to the fact that they are usually chaperoned by various carriers such as RNA-binding proteins like AGO2 [86] or lipoprotein complexes [87], or are packed in EVs [88]. To date, miRNAs have been shown to change significantly throughout the course of many diseases and physiological conditions, including pregnancy disorders [89]. Therefore, they have emerged as potent biomarkers for the diagnosis and prognosis of a disease in a minimally invasive way.

The characteristic composition of circulating miRNAs has been shown in several animals, including domestic animals, during normal and complicated pregnancies. For example, miR-26a levels were higher in 3-week pregnant heifers compared with sham-inseminated counterparts [90]. Broadly conserved miRNAs (e.g., let-7c, miR-143) were found to be signatures of pregnancy in cows; however, elevated levels of these miRNAs were observed during late pregnancy [91,92]. Interestingly, circulating EV-derived miRNAs (e.g., miR-125b-2, let-7c) were associated with increased embryonic mortality on day 17 of pregnancy in cows [93]. However, EV-derived and broadly conserved miR-195 was maintained at a high level in pregnant mares on day 11 [94].

Recently, we detected three pregnancy-specific circulating miRNAs in maternal circulation immediately after initiation of implantation [41]. Increased levels of circulating miRNAs, previously reported to be expressed in either conceptuses (miR-26a and miR-125b [22]) or pregnant endometria (miR-23b [23]), were detected on day 16 of pregnant pigs. Common biological processes and pathways affected by the three miRNAs (e.g., Wnt/beta-catenin, TGF-beta, and FGF signaling), as well as the number of targets (e.g., *IFNG*, *LIFR*, *FGFR2*, *TGFBR1*, *ESR1*, *SP1*) with important roles during pregnancy, were identified *in silico*, supporting the concept that we may indeed observe signs of ongoing embryo–maternal crosstalk in the circulation of the pregnant pig. Further results showed a plethora of reproductive status-dependent miRNAs, allowing to distinguish between pregnant and non-pregnant animals on days 16 and 20 of pregnancy [42]. Interestingly, serum samples were enriched with miRNAs involved in processes occurring during early pregnancy, such as angiogenesis, embryonic cell proliferation, and differentiation. In addition, analyzed serum samples contained both EVs and AGO2 proteins, both involved in miRNA transportation and cell-to-cell communication. Our results support the possibility of using circulating miRNAs that differ in abundance between cyclic and pregnant pigs as potential indicators of reproductive status during breeding management.

## 8. Concluding Remarks and Future Directions

**Figure 3:**
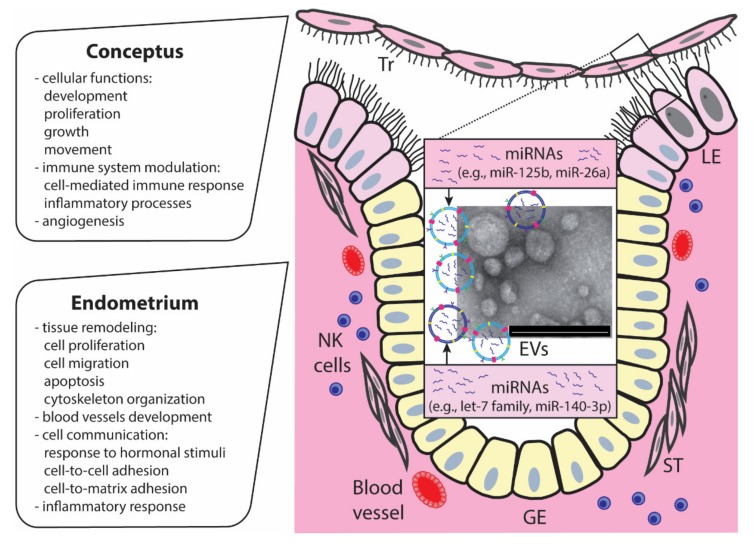
Peri-implantation events are governed by various biological molecules, including miRNAs produced by the endometrium and conceptus in pigs. miRNAs carried by EVs participate in reciprocal communication between the conceptus and endometrium, regulating the expression of genes involved in several pathways crucial for successful implantation. Tr, trophoblast; LE, luminal epithelium; GE, glandular epithelium; ST, stroma; NK cells, natural killer cells. Scale bar, 200 μm.

Clearly, miRNAs can be now recognized as powerful gene expression regulators during the peri-implantation period in livestock, such as pigs. We and others have clearly shown that miRNAs are present at the embryo–maternal interface and can target genes supporting early pregnancy events, such as embryo development and implantation (Figure 3). However, recent experiments have mostly focused on transcriptomic screening and *in silico* miRNA–mRNA interaction predictions, and only a few have shown experimentally the impact of particular miRNAs on the trophoblast or endometrial function. The latter observations revealed, however, the complexity of miRNA-mediated gene regulation during early pregnancy in pigs. Still, our understanding of when and how miRNAs can exert regulatory effects on transcription of the embryo–maternal interface is very limited. Deeper investigation is needed to understand which factors contribute to the activity of miRNAs in trophoblasts and endometria, including cell-specific and subcellular location, miRNA and target abundance, miRNA–mRNA binding affinity, miRNA cluster cooperation, and many more related to the cellular context-dependent response.

Recent discoveries of EVs released by both the endometrium and embryos revealed an additional layer to cell-to-cell communication during the peri-implantation period. The conducted research gives an initial picture of the possible embryo–maternal interactions involving EVs. However, many aspects have not yet been investigated, including biogenesis of EVs and packing of the specific molecular cargo in response to different stimuli. Thus far, no mechanism has been described to guide the EVs to deliver selected cargo to target cells at the embryo–maternal interface.

Overall, the latest research definitely extends our knowledge about the expression and role of miRNAs in the luteal tissue. However, more efforts should be made to characterize miRNAs that can be unique/common among different animal species or specific for CL cells, the role of specific miRNA–mRNA interactions, and the regulation of miRNA expression by luteotrophic or luteolytic factors at the crucial and decisive moment when live embryos are present in the uterus.

Cortez and coworkers [95] hypothesized that miRNAs can function as the “oldest” hormones secreted into blood plasma, and in this way, they can exert their effects onto distant parts of the body. Although it seems likely that miRNAs overexpressed during the peri-implantation period are released into the bloodstream from reproductive tissues and developing conceptuses, direct evidence for this or its mechanism is still missing.

Pregnancy monitoring using miRNAs is of particular interest due to often-occurring difficulties in diagnosing complications in the early stages combined with their potentially catastrophic consequences. Research related to detecting and understanding the role of particular miRNAs during the peri-implantation period is noteworthy for animals with high early embryonic mortality, such as pigs. In any species, earlier diagnosis allows for earlier intervention, leading to favorable outcomes.

## Figures and Tables

**Table 1:** Examples of microRNAs (miRNAs) detected in pregnant pigs.

Source of MiRNAs		Examples of Detected MiRNAs	Ref.
**Embryo**	8 cell	miR-17, -125a-5p, -125b, -128, -205	[17]
	blastocyst	miR -92a, -129-5p, -205, -210, -302a	[17]
	day 10	miR-371-3p, -455, -449a, -634, -940, -1193-3p	[22]
		miR-7, -302b, -302d, -371-5p, -378	[38]
	day 12	miR-96, -138, -152, -483-5p, -765	[22]
		miR-301, -467, -4057	[34]
	day 14	miR-10a, -21, -23b, -200a, -301a, -574	[34]
	day 16 (trophoblast)	miR-26a, -27a, -148a	[22]
	day 20 (trophoblast)	miR-125b, -199a-3p, -199a-5p	[22]
		miR-15b-5p, -18a, -20a-5p, -126-5p, -155-5p, -221-5p	[32]
	day 25 (placenta)	miR-17, -18a, -19a, -20a, -92a	[36]
	day 30 (placenta)	miR-17, -106a, -107, -345-5p, -615-3p	[35]
	day 50 (trophoblast/placenta)	let-7f-5p, miR-150, -221-5p,-222,	[32]
		let-7a/b/c/d/e/f/i, -23a, -24, -27a/b, -29a, -30a/e, -141, -205	[36]
**Endometrium**	day 12	let-7f/g/i, miR-10b/d, -143-3p	[38]
	day 15	let-7b/c/i, miR-125a-3p, -135a*, -140-3p, -149, -181c/d, -200c, -361, -494, -542-5p	[37]
	day 16	miR-23b, -127, -411, -449a	[23]
	day 20	miR-1, -10b, -27a, -30d -101-1, -126, -143, -146b, -193, -193b-3p, -205, -574	[23]
		miR-21, -22-3p, -27a -29a, -30b-5p, -30d, -30e-5p, -149, -183, -191, -205, -296, -323, -362, -432-3p,-503, -574, -4335, -4339	[33]
	day 26	miR-15b, -17-5p, -18a, 20a, 30a-3p/5p, -92b, -106a, -126, -132, -221, -222	[37]
	day 50	miR-30c/e, -215, -411, -487b	[37]
**EVs^1^**	ULFs^2^ from gestational day 14 and 16	miR-26a, -125b	[22]
	CM^3^ of porcine trophectoderm cells	miR-15b, -16, -17-5p, -20a, -126-5p, -150, -155-5p, -221-5p	[39]
**Ovary**	CL from gestational day 14	miR-21a-3p, -345-3p, -371-5p, -4334-5p, -9788-3p, -9840-3p, -9850-5p	[40]
**Serum**	gestational day 16 and 20	let-7a/b/c/f, miR-10a, -23b, -26a, -30b-5p, -125b, -143-3p	[41,42]

^1^ EVs—extracellular vesicles; ^2^ ULFs—uterine luminal flushings; ^3^ CM—conditioned media

## Abbreviations

3′ UTR	Three prime untranslated region
5′ UTR	Five prime untranslated region;
ACVR2B	Activin receptor type-2B
AGO1-4	Argonaute RISC components 1-4
AKR1C1	Aldo-keto reductase family 1 member C1
CDC42	Cell division cycle 42
CL	Corpus luteum
CREB	Cyclic AMP-responsive element-binding protein
DGCR8	DiGeorge syndrome critical region gene 8
DICER1	Dicer 1, ribonuclease III
DROSHA	Drosha, ribonuclease III
E2	Estradiol-17beta
EDN1	Endothelin 1
ESCRT	Endosomal sorting complex required for transport
ESR1	Estrogen receptor 1
ESR2	Estrogen receptor 2
EVs	Extracellular vesicles
FGF	Fibroblast growth factor
FGFR2	Fibroblast growth factor receptor 2
FOS	Fos proto-oncogene
IFNG	Interferon gamma
JUN	Jun proto-oncogene
KCNMA1	Potassium calcium-activated channel subfamily M Alpha 1
LIF	Leukemia inhibitory factor
LIFR	Leukemia inhibitory factor receptor
miRNA	MicroRNA
NR4A1	Nuclear receptor subfamily 4 group A member 1
OXTR	Oxytocin receptor
P4	Progesterone
PGE2	Prostaglandin E2
PGR	Progesterone receptor
PTGER2	Prostaglandin E receptor 2
PTGS2	Prostaglandin-endoperoxide synthase
RAB	RAS-related protein GTPases
RISC	RNA-Induced Silencing Complex
SIRT1	Sirtuin 1
SNP	Single nucleotide polymorphism
TARBP 2	HIV-1 transactivation response (TAR), RNA- binding protein 2
TGF-beta	Transforming growth factor beta
TGFBR1	Transforming growth factor beta receptor 1
TNRC6A	Trinucleotide repeat containing 6A
VEGFR1	Vascular endothelial growth factor A receptor 1
XPO5	Exportin-5
